# Attachment representations in pre‐adolescents at familial high risk of schizophrenia or bipolar disorder and population‐based controls—Characteristics of attachment from middle childhood to pre‐adolescence, and its relation to parental functioning and child mental disorder

**DOI:** 10.1002/jcv2.12274

**Published:** 2024-09-12

**Authors:** Mette Falkenberg Krantz, Maja Gregersen, Lotte Veddum, Carsten Hjorthøj, Åsa Kremer Prøsch, Jessica Ohland, Julie Marie Brandt, Sinnika Birkehøj Rohd, Christina Bruun Knudsen, Anna Krogh Andreasen, Nicoline Hemager, Aja Greve, Ole Mors, Merete Nordentoft, Elia Psouni, Anne A. E. Thorup

**Affiliations:** ^1^ CORE‐ Copenhagen Research Center for Mental Health Mental Health Center Copenhagen Capital Region of Denmark Copenhagen University Hospital Copenhagen Denmark; ^2^ Research Unit at Child and Adolescent Mental Health Center Copenhagen Capital Region of Denmark Copenhagen Denmark; ^3^ iPSYCH ‐The Lundbeck Foundation Initiative for Integrative Psychiatric Research Aarhus Denmark; ^4^ The Psychosis Research Unit Aarhus University Hospital – Psychiatry Aarhus Denmark; ^5^ Faculty of Health and Medical Services Department of Clinical Medicine Aarhus University Aarhus Denmark; ^6^ Department of Public Health Section of Epidemiology University of Copenhagen Copenhagen Denmark; ^7^ Faculty of Social Sciences Department of Psychology University of Copenhagen Copenhagen Denmark; ^8^ University of Copenhagen—Faculty of Health and Medical Sciences Institute of Clinical Medicine Copenhagen Denmark; ^9^ Department of Psychology Lund University Lund Sweden

**Keywords:** attachment, bipolar, high risk studies, risk factors, schizophrenia

## Abstract

**Background:**

Development of secure attachment is crucial to establish and maintain healthy relationships with others throughout life. For parents with schizophrenia or bipolar disorder, challenged parental functioning may compromise the sensitive caregiving needed to establish secure child attachment. We aimed to examine pre‐adolescent attachment, predictors related to caregiving, and middle childhood attachment predictors of pre‐adolescent mental disorders.

**Methods:**

In a population‐based nationwide cohort of 522 children of parents with schizophrenia or bipolar disorder and population‐based controls, The Secure Base Script Test was used to assess attachment security at age 11 (*N* = 409). Parental caregiving and functioning were assessed with the Personal and Social Performance Scale and MC‐HOME (age 7) and child mental disorder with K‐SADS‐PL (age 7 and 11). Story Stem Assessment Profile was used for age 7 attachment.

**Results:**

We found no differences between risk and control groups in prevalence of pre‐adolescent secure attachment. Parental level of functioning and attachment security at age 7 significantly predicted more rich secure base content at age 11. Level of age 7 disorganization significantly predicted presence of mental disorder at age 11.

**Conclusions:**

Overall, attachment of children at familial risk of schizophrenia or bipolar disorder did not differ from that of controls. Instead, parental functioning in middle childhood predicts pre‐adolescent attachment, and may therefore serve as a focus for supporting healthy attachment development. Middle childhood disorganization might serve as a predictor of pre‐adolescent mental disorder if other studies confirm our findings, and awareness hereof may be relevant for guiding intervention to support mental development.

## Graphical abstract

Pre‐adolescent attachment, predictors related to caregiving, and middle childhood attachment predictors of pre‐adolescent mental disorders was assessed in a cohort of children of parents with schizophrenia or bipolar and population‐based controls. ‐ We found no differences between risk and control groups in prevalence of pre‐adolescent secure attachment. ‐ Parental level of functioning at age 7 significantly predicted more rich secure base content at age 11, and level of age 7 disorganization significantly predicted presence of a mental disorder at age 11.

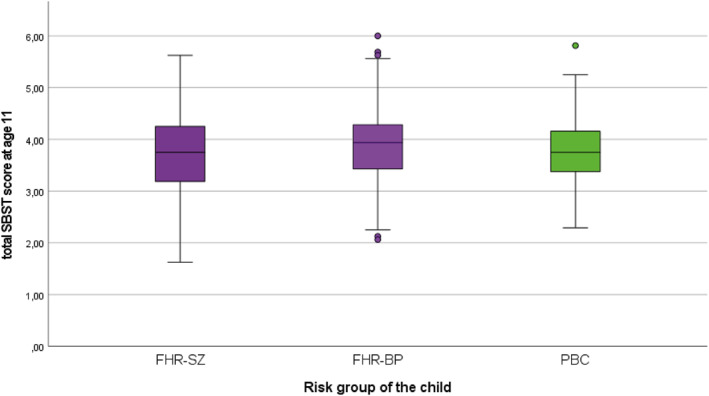

**Key points**

- Parental mental disorder can pose a risk to attachment development but studies after infancy have lacked
- We examined attachment across two time points from middle childhood to pre‐adolescence in children of parents with schizophrenia or bipolar disorder and controls
- We found no group differences in attachment
- High parental functioning predicted later secure attachment, and disorganization predicted higher risk of mental disorder, across middle childhood and pre‐adolescence
- Awareness of low parental functioning and child disorganization is needed to support attachment development.

## INTRODUCTION

Attachment theory states that a child from its first years of life depends on developing a stable relationship with at least one caregiver in order to later establish and maintain healthy social and emotional relationships with others (Bowlby, 1969; Cassidy, 2016). Secure attachment is created through loving, sensitive, and lasting relationships with caregivers who respond towards child distress in sensitive and supportive manners. Caregivers thereby help their children to develop positive self‐representations and a sense of being worthy of love (Bohlin et al., 2000; Bowlby, 1988; Bretherton, 1995; Psouni et al., 2015). Attachment is believed to later influence expectations and internal representations of relationships with caregivers, friends, and partners (Ann Easterbrooks & Abeles, 2000; Fraley, 2010).

Attachment in children is typically categorized as Secure, Avoidant, Ambivalent and Disorganized (Ainsworth et al., 1979; Greenberg et al., 1990). Studies have found disorganized attachment to be a risk factor for mental illness and externalizing symptoms (Carlson, 1998; Granqvist et al., 2017; Groh et al., 2017), and insecure attachment to predict internalizing and externalizing problems (Belsky et al., 2010; Brumariu & Kerns, 2010; Fearon et al., 2010; Madigan et al., 2013) and mental illness (Groh et al., 2012). Studies have found associations between insecure attachment and lack of social competence and self‐representations, poor relations to peers, poor ability to cope with stress and poor emotion regulation (Ann Easterbrooks & Abeles, 2000; Delgado et al., 2022; Groh et al., 2014; Pallini et al., 2019; Psouni & Apetroaia, 2014; Psouni et al., 2015; Sroufe, 2005). Insecure attachment style, including disorganization which can be secure as well as insecure, is thus a risk factor for a range of social and behavioral problems and mental illness (James et al., 2018; Lyons‐Ruth et al., 2013; Paul, 2007).

Severe parental mental illness such as schizophrenia, bipolar disorder, and severe depression compromises the ability to provide the sensitive and stable interaction required to promote the child's development of attachment security (Anke et al., 2020; Carter et al., 2001; Davidsen et al., 2015). In families where one or both parents are affected by severe mental illness, previous studies have found increased prevalence of negative interaction and communication style, mood swings, distress, low parenting capacity, poor mentalization, and low daily functioning (Brockington et al., 2011; Chernomas et al., 2000; Duncan & Reder, 2000; Goossens et al., 2008; Riordan et al., 1999; Rosa et al., 2008; Vance et al., 2008; Wan et al., 2008). While studies have dealt with such risk factors for child attachment, only few have examined attachment in offspring of parents with severe mental illness. Therefore, our understanding of attachment among these children is sparse, especially after infancy (Davidsen et al., 2015). For children of mothers with schizophrenia, two studies found elevated rates of insecure attachment at infancy (mean age 12.5 months and 1 year) (D’Angelo, 1986; Näslund et al., 1984) while another did not find elevated rates (age range 0–2.5 years) (Sameroff et al., 1982). A study of adult offspring raised by parents with schizophrenia found attachment problems resulting in difficulties in forming secure relationships, including problems with trust and intimacy (Duncan & Browning, 2009). For children of parents with bipolar disorder, two studies of infants/toddlers (age range 2–3 years) and adolescents (age range 12–17) found elevated rates of insecure attachment (Erkan et al., 2015; Zahn‐Waxler et al., 1984) while a study of offspring aged 7–20 did not (Doucette et al., 2013). A study of children above 16 years of age found increased rates of anxious/ambivalent attachment compared with controls (Kökçü & Kesebir, 2010). Studies examining both schizophrenia and bipolar disorder found no correlation between parental illness or functioning and disrupted mother‐child interaction (at 4 months) (Nyström‐Hansen et al., 2019), and no differential attachment across groups (at mean age 7.8) (Gregersen et al., 2023). In pre‐adolescence, attachment studies of offspring to parents with severe mental illness are few, and assessments are challenged by lack of agreement in the literature about the best approach to measure attachment in middle childhood and pre‐adolescence (Jewell et al., 2019). One widely used approach is to assess the child's attachment event schemas (scripts), which are considered to be implicit components of the child's attachment representations that encapsulate knowledge and expectations about what happens in times of distress (Fivush, 2006; Psouni & Apetroaia, 2014; Waters & Waters, 2006). Examples of such assessments measuring attachment scripts in childhood and adolescence are The Story Stem Assessment Profile (SSAP) (Hodges et al., 2014) and The Secure Base Script Test (SBST) (Psouni & Apetroaia, 2014). Examining pre‐adolescent attachment representations among children of parents diagnosed with a severe mental illness is important since it is a time of consolidation of internal working models (Jones et al., 2018; O’Shaughnessy, 2023) and additionally, often challenges the caregiver‐child relation (Morris et al., 2021).

The metanalytical evidence of only moderate stability of attachment from infancy to early adulthood (Bateson, 2023; Belsky, 1997; Pinquart et al., 2013) suggests that the child's attachment representations, and thus sense of security, continue to be malleable throughout childhood. Since previous studies of children of parents with severe mental illness have mainly been cross‐sectional, and since general population studies have shown correlations between insecure attachment and later mental disorder, for example, ADHD (Cavicchioli et al., 2023), a two time‐point examination of attachment and outcomes indicating negative mental development, as well as an inclusion of caregiving predictors, could generate knowledge of relevance for intervention among these children, who are already at increased risk of developing mental illness (Rasic et al., 2014; Uher et al., 2023).

### Aims and objectives

The scope of this study was to examine pre‐adolescent attachment at age 11, caregiving quality as predictor of attachment, and potential attachment predictors of mental disorders from middle childhood to pre‐adolescence in a cohort of children of parents with schizophrenia or bipolar disorder and population‐based controls. The specific questions asked were:

1. whether attachment at age 11 differed amongst children of parents with schizophrenia or bipolar disorder and controls,
2. whether high levels of insecure and disorganized attachment at age 7 predicted less secure attachment at age 11, and
3. whether high levels of insecure and disorganized attachment at age 7 predicted presence of a mental disorder at age 11.
  Further, because of the importance for attachment development of parental ability and engagement in interaction and support of the child, we examined
4. whether caregiver level of functioning and level of stimulation and support provided in the home at age 7 predicted child attachment score at age 11.

Based on baseline study findings (Gregersen et al., 2023), no overall group differences were expected.

We hypothesized that high levels of insecure and disorganized attachment at age 7 would predict less secure attachment and would predict mental disorder at age 11. We likewise hypothesized that high levels of secure age seven attachment would decrease risk of insecure attachment and decrease presence of a mental disorder at age 11. Further, we hypothesized that low caregiver level of functioning and lower levels of stimulation and support provided at home would predict less secure attachment at age 11.

## MATERIALS AND METHODS

### The cohort

The Danish High Risk and Resilience Study is a population‐based, two‐site, prospective cohort study assessing 522 same‐aged children of parents with a schizophrenia spectrum or schizoaffective disorder (denoted FHR‐SZ for familial high risk of schizophrenia, *N* = 202) or bipolar disorder (denoted FHR‐BP for familial high risk of bipolar disorder, *N* = 120) and population‐based controls (denoted PBC, *N* = 200) with no such diagnoses (Thorup et al., 2015). The children in the PBC group were matched to the children at FHR‐SZ on age, sex, and municipality. The children at FHR‐BP were unmatched but did not differ from the other two groups on age or sex. The caregiver who lived with the child and spent most time taking care of the child was defined as the *primary caregiver*, and this could therefore be the ill parent, the co‐parent, or another caregiver. Baseline assessments were conducted at age 7 (the VIA 7 Study) and follow‐up assessments were conducted at age 11 (the VIA 11 Study, see Thorup et al., 2018 for study protocol). Assessors were medical doctors, psychologists, and research nurses. All received formal training in the assessment battery prior to study initiation. Assessments took place at research facilities in Copenhagen and AÅrhus, Denmark. The child assessors were blinded to the risk status of the child. The study data was collected and managed using REDCap (Research Electronic Data Capture) (Harris et al., 2019).

### Measures at age 7

The Story Stem Assessment Profile (SSAP), seven‐story‐version, described in detail elsewhere (Gregersen et al., 2023), was used for attachment script assessment at age 7, with coding by raters with accreditation from the Anna Freud National Center for Children and Families. It consists of four continuous constructs named Disorganization, Security, Insecurity and Defensive Avoidance. The SSAP lacks validation but has been shown to have acceptable to good internal consistency across its four constructs (Cronbach's Alpha for indicators of Security = 0.78, Insecurity = 0.73, Defensive Avoidance = 0.72 and Disorganization = 0.87) at our baseline study, and further, correlations between Insecurity and Disorganization scores and higher likelihood of a concurrent mental disorder have been identified (Gregersen et al., 2023). Child mental disorder (lifetime, excluding elimination disorders (Ellersgaard et al., 2018)) used for controlling of baseline presence, and global functioning was assessed through the use of the Schedule for Affective Disorders and Schizophrenia for school‐age children – present and lifetime version (K‐SADS‐PL) (Kaufman et al., 1997). Caregiver level of functioning was assessed with the semi‐structured interview Personal and Social Performance Scale (PSP) (Morosini et al., 2000; Thorup et al., 2016) and the level of stimulation and support provided in the child's home was assessed with the semi‐structured interview Middle Childhood HOME Inventory (MC‐HOME) (Elardo & Bradley, 1981; Gantriis et al., 2019). Information concerning caregiver civil status, level of education, children living out of home, and child and family support provided was obtained in anamnestic interviews (Thorup et al., 2015). For in‐depth description of measures at age 7, see (Thorup et al., 2015).

### Measures at age 11

The SBST is a narrative‐based assessment of attachment scripts, defined as rudimentary attachment related representations which capture the child's beliefs and expectations about interactions with important others when a serious difficulty is encountered, reflecting the child's probable experiences and behaviors in such situations (Psouni & Apetroaia, 2014). The SBST has been found to have high internal consistency, high interrater reliability, and construct validity with well‐known attachment measures (Psouni & Apetroaia, 2014) (See Supplementary Text for further details). Because of the increasing significance of close friends from middle childhood and pre‐adolescence, the measure includes both attachment figures and best friends as important others. In this narrative‐based assessment, the child is asked to create stories using prompt outlines eliciting attachment‐related situations with parents (“Math Test” and “Accident”) and with a best friend (“Trouble at school” and “Moving”) (Psouni & Apetroaia, 2014). Storylines consist of 12 words, suggesting that the main character encounters a difficulty, possible interaction, and possible resolution. The outlines were presented one at a time in random sequences. Upon controlling that the child was able to read the prompt words, instructions were given to create “the first story that comes to mind, but a good and long story, with many details”. The task took about 20 min to complete. Stories were tape‐recorded, transcribed, and scored according to SBST scoring guidelines (Psouni & Apetroaia, 2013), using a seven‐point scriptedness scale reflecting amount of secure base/safe haven knowledge in the child's narrative. Higher scores reflect richer secure base content including descriptions of emotional states and interactions. Stories were scored by two trained coders (LV and ÅKP, trained by EP alongside initiation of data collection) and a senior trained coder (EP) (25% of the material). Assessors involved in scoring were blinded for risk status. Internal consistency based on 12.2% (50 cases) of the material (*N* = 18 for FHR‐SZ, *N* = 4 for FHR‐BP, *N* = 28 for PBC) was excellent with Cronbach's Alpha values of 0.96–0.97 (mean value = 0.96) and likewise, the ICC across coders was excellent, also with an average of 0.96 (Table S1).

A total score was produced by averaging the scores across the four stories from each child. Parent‐child scores were calculated averaging the “Accident” and “Math Test” scores, and child‐child scores by averaging the “Trouble at School” and “Moving” scores. Further, word count estimates of words used in the stories were calculated.

Data concerning caregiver characteristics and support provided by the municipality was obtained through anamnestic interviews. Caregiver level of functioning, child mental disorder (lifetime, excluding elimination disorders, transient and unspecified tics, and specific phobias (Gregersen et al., 2023) and global functioning were assessed as described for age 7. For in‐depth description of measures, see (Thorup et al., 2018).

## STATISTICAL METHODS

In accordance with baseline study findings of low kappa for the SSAP Defensive Avoidance construct, this construct was removed from our models (Gregersen et al., 2023).

The total age 11 attachment score was dichotomized by defining a cut‐off below 4 (=3.9999) to denote a rudimentary but clearly secure base script, in line with previous studies that have found support for this dichotomy (Di Folco et al., 2017; Psouni & Apetroaia, 2014; Psouni et al., 2015). The dichotomized score was used to illustrate secure versus insecure scripts across groups in Table 2, and the continuous variable was used for all other analyses in order not to lose information from dichotomization.

Pearson's correlations were assessed between word length and SBST score, between age 7 and 11 attachment and between placement out of home and age 7 attachment. Stem‐and‐leaf plots were used to assess characteristics of participants with the highest and lowest attachment scores. For analyses of attachment across groups (Table 2), Bonferroni post hoc correction was used for pairwise comparisons.

General linear models (ANCOVAs) were used to assess relationships between attachment at ages 7 and 11, and to assess how each age 7 attachment construct level predicted attachment at age 11, and how parental level of functioning and at‐home level of stimulation and support at age 7 predicted age 11 attachment. ANCOVAs were also used to assess relationships between age 7 attachment and age 11 mental disorder, adjusted for presence of age 7 mental disorder. In contrast to Table 1 data which was run only on age 11 participants with available attachment data as this was our main outcome, ANCOVAS were run on the full dataset of 522 children in order to include all children with available data concerning the variables of interest, therefore numbers in analyses differ depending on the outcome variable. Analyses were run in combined models for each outcome. When an interaction was present between risk group and the variable of interest, the analysis was presented separately. Where no interactions were present, the interaction term was removed from the model. All analyses were performed using SPSS Statistics version 25.

**TABLE 1:** Characteristics of 409 children of parents with schizophrenia or bipolar disorder and population‐based controls at ages 7 and 11.

*N* = 409^a^	FHR‐SZ (*N* = 157)	FHR‐BP (*N* = 92)	PBC (*N* = 160)	*p*‐value	FHR‐SZ versus PBC, *p* (CI)	FHR‐BP versus PBC, *p* (CI)	FHR‐SZ versus FHR‐BP, *p* (CI)
Sex, female, *N* (%)	79 (50.3%)	41 (44.6%)	75 (46.9%)	0.66	–	–	–
Age 7							
Living in inadequate home environment at age 7, *N* (%)^b^	31 (19.9)	9 (9.9)	8 (5.0)	**<0.001**	**<0.001** (−0.22; −0.08)	0.25 (−0.03; 0.13)	**0.02** (−0.18; −0.02)
Levels of stimulation and support provided at home, age 7 score^b^	45.49 (6.31)	46.92 (4.64)	49.06 (4.55)	**<0.001**	**<0.001** (−4.75;‐ 2.40)	**0.002** (0.77; −3.51)	**0.04** (−2.81; −0.06)
Any axis 1 diagnosis, *N*(%)	78 (50.3)	47 (51.1)	36.9 (32.1)	**0.03**	**0.02** (0.02; 0.24)	**0.03** (−0.27; −0.01)	0.91 (−0.14; 0.12)
Level of functioning of the primary caregiver, mean (SD)	73.92 (14.43)	75.45 (13.78)	84.43 (9.23)	**<0.001**	**<0.001** (−13.29; −7.75)	**<0.001** (−12.20; −5.78)	0.35 (−4.76; 1.70)
Age 11							
Age, mean (SD)	11.96 (0.26)	11.94 (0.21)	11.92 (0.22)	0.40^c^	–	–	–
Child's level of functioning^d^	64.64 (15.54)	69.21 (14.10)	75.68 (13.73)	**<0.001**	**<0.001** (−14.25;−7.83)	**<0.001** (10.21; −2.73)	**0.02** (−8.32;−0.82)
Lives with index^e^	112 (55.4)	69 (57.5)	170 (85.0)	**<0.001**	**<0.001** (−0.32; −0.15)	**<0.001** (0.21; 0.41)	0.13 (−0.02; 0.18)
Child lives with single caregiver at age 11, *N* (%)	48 (30.6)	38 (41.3)	22 (13.8)	**<0.001**	**<0.001** (0.07; 0.26)	**<0.001** (−0.39; −0.16)	0.58 (−0.22; 0.00)
Index parent is single caregiver	21 (13.4)	12 (13.0)	14 (8.8)	0.40	–	–	–
Living out of home	16 (10.2)	<5	<5	**0.00**	**<0.001** (0.06; 0.15)	0.67 (−0.04; 0.06)	**<0.001** (0.04; 0.14)
Has received supplementary support for family, *N* (%)	53 (33.8)	27 (29.3)	16 (10.1)	**<0.001**	**<0.001** (0.15; 0.33)	**<0.001** (−0.30; −0.09)	0.42 (−0.06; 0.15)
Has received supplementary support for child, *N* (%)	81 (51.6)	41 (44.6)	40 (25.2)	**<0.001**	**<0.001** (0.16; 0.37)	**0.002** (−0.32; −0.07)	0.26 (−0.05; 0.19)
Any axis 1 diagnosis age 11, *N* (%)	100 (64.5)	59 (64.1)	77 (48.1)	**<0.01**	**<0.01** (0.06; 0.27)	**0.01** (−0.29; −0.03)	0.95 (−0.12; 0.13)
Level of functioning of the primary caregiver, mean (SD)	69.89 (16.88)	72.37 (15.62)	83.50 (10.25)	**<0.001**	**<0.001** (−16.79; −10.43)	**<0.001** (−14.83; −7.43)	0.19 (−6.18; 1.23)

*Note:* Bold values indicate the significant values at the 0.05 level.

Abbreviations: FHR‐BP, familial high risk of bipolar disorder; FHR‐SZ, familial high risk of schizophrenia; PBC, population‐based controls.

^a^ As the SBST age 11 attachment score was our main outcome, Table 1 cohort characteristics analyses were restricted to the 409 children who participated in this assessment.

^b^ Assessed with Middle Childhood Home Inventory (MC‐HOME).

^c^ One‐way ANOVA. Age is highlighted for age 11 assessment as it is our main outcome.

^d^ Assessed with Children's Global Assessment Scale (CGAS).

^e^ Index parent denotes the parent who was identified in registers as the ill parent. In the control group, it denotes the matched non‐ill parent of the same sex.

## RESULTS

A total of 409 pre‐adolescents (FHR‐SZ = 157, FHR‐BP = 92 and PBC = 160, mean (SD) age 11.9 (0.23)) participated in the age 11 SBST assessment (Table 1 and Figure S1). A total of 510 children had data on either age 7 or 11 attachment (baseline *N* = 482 for SSAP as reported in (Gregersen et al., 2023)).

For dropout analyses, inspection of data including word counts, outlier characterization, and attachment in relation to placement out of home, see Supplementary Text and Table S2–S4.

### Cohort characteristics

Across groups, the children did not differ in terms of sex or age (Table 1). At age 7, more children at FHR‐SZ lived in inadequate home environments compared with controls. Both high risk groups had lower levels of stimulation and support compared with PBC. Higher proportions among high risk groups fulfilled criteria for a psychiatric diagnosis, and primary caregivers in the high risk groups had lower levels of functioning compared with controls. At age 11, lower proportions among high risk groups were found to live with the ill parent as compared with the matched parent in the control group, and higher proportions of the two high risk groups lived with a single caregiver. A higher proportion of FHR‐SZ children lived away from their biological parents, and higher proportions among FHR children had received support for the family or for the child. Higher proportions of FHR children fulfilled criteria for a psychiatric diagnosis at age 11, and parents belonging to the FHR‐SZ and FHR‐BP groups had lower levels of functioning compared with controls (Table 1).

### Does attachment at age 11 differ among children at familial high risk compared to controls?

**FIGURE 1:**
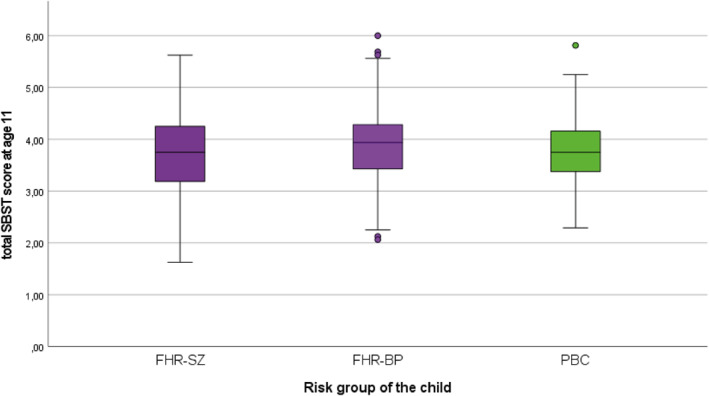
Total attachment scores among children of parents with FHR‐SZ, FHR‐BP or population‐based controls assessed with the secure base script test at age 11. FHR‐BP, familial high risk of bipolar disorder; FHR‐SZ, familial high risk of schizophrenia.

We found no overall differences between the FHR‐SZ, FHR‐BP and PBC groups when assessing attachment SBST sub‐scores concerning parent‐child relation or relation to friends, nor when assessing SBST total scores (Table 2 and Figure 1). No difference was found between the three groups when assessing how many children had a secure base script at age 11 (Table 2). Explorative dichotomization with a lower and thus, less conservative cutoff of 3.5 returned a slightly higher proportion of secure base scripts among controls compared to the FHR‐SZ group (FHR‐SZ, N (%) = 140 (69.3), FHR‐BP, N (%) = 90 (75.0), PBC, N (%) = 148 (74.0)), but group differences were not significant (data not shown). Further, no difference was found between the three groups with regard to word length of the produced stories in the SBST test (Table 2).

**TABLE 2:** Subscores, total scores, secure base script and word count in the secure base script test among children of parents with schizophrenia, bipolar disorder, and population‐based controls.

*N* = 409	FHR‐SZ (*N* = 157)	FHR‐BP (*N* = 92)	PBC *N* = 160)	*p*‐value	FHR‐SZ versus PBC	FHR‐BP versus PBC	FHR‐SZ versus FHR‐BP
Parent‐child subscore^a^, mean (SD)	3.64 (0.82)	3.74 (0.84)	3.77 (0.75)	0.35	–	–	–
Friend‐friend subscore^a^, mean (SD)	3.77 (0.89)	4.01 (0.96)	3.85 (0.70)	0.09	–	–	–
Total SBST score, mean (SD)	3.70 (0.76)	3.88 (0.81)	3.81 (0.64)	0.15	–	–	–
Secure base script, *N* (%)	64 (40.8)	43 (46.7)	60 (37.5)	0.36	–	–	–
Word count for all four SBST stories, mean (SD)^b^	385.39 (285.60)	477.43 (380.43)	440.06 (387.36)	0.12	–	–	–

Abbreviations: FHR‐BP, familial high risk of bipolar disorder; FHR‐SZ, familial high risk of schizophrenia; PBC, population‐based controls.

^a^ *N* = 407 as *N* for FHR‐SZ = 155.

^b^ *N* = 405 since only stories which could be scored with a full word count for all stories are included.

### Does attachment, caregiver level of functioning and level of stimulation and support at home at age 7 predict attachment at age 11?

Using general linear models of age 7 attachment and age 11 attachment, we found that a high SSAP security construct at age 7 predicted a high SBST attachment score at age 11, indicating more rich secure base content (Table 3). Interaction was found with both high risk groups and the security construct. The level of stimulation and support provided at home at age 7 did not predict attachment at age 11, nor did the SSAP insecurity and disorganization constructs at age 7. Parental level of functioning at age 7 predicted age 11 attachment although the variance explained was little (Table 3). Interaction was found with the FHR‐BP group for parental level of functioning and was therefore rerun in a separate analysis, but when run in the separate model, this interaction was no longer significant. An explorative analysis of correlation between caregiver's level of functioning at the child's age 11 and age 11 attachment score likewise showed a strong correlation (*p* < 0.001, Pearson's *r* = 0.108) (data not shown).

**TABLE 3:** General linear model presenting the degree to which low attachment score, levels of stimulation and support provided at home at the child's age 7, and parental level of functioning is predictive of low attachment at age 11.

	Type III sum of squares	df	Mean square	*F*	Significance	Partial eta squared
Risk group	1.01	2	0.50	0.92	0.40	0.01
Level of stimulation and support at home at age 7	0.89	1	0.89	1.62	0.20	0.004
Attachment insecurity construct at age 7^a^	0.86	2	0.86	1.56	0.21	0.004
Attachment disorganization construct at age 7^a^	0.90	1	0.90	1.64	0.20	0.004
Total	5606.58	378	–	–	–	–
Corrected total	207.19	377	–	–	–	–
Risk group × parental level of functioning at age 7^b^	3.10	2	1.55	2.97	0.05	0.02
Parental level of functioning at age 7	4.14	1	4.14	7.91	**0.01**	0.02
Total	6006.32	405	–	–	–	–
Corrected total	216.55	404	–	–	–	–
Risk group × attachment security construct at age 7^a^ ^,^ ^c^	5.34	2	2.67	5.44	**0.01**	0.03
Attachment security constuct at age 7^a^	19.29	1	19.29	39.35	**<0.001**	0.10
Total	5647.16	381	–	–	–	–
Corrected total	207.79	380	–	–	–	–

*Note:* Bold values indicate the significant values at the 0.05 level.

^a^ Assessed with the Story Stem Assesment Profile (SSAP).

^b^ Separate analysis due to significant interaction with FHR–BP risk group (*p* = 0.03) when run in combined analysis. When analyzed separately, the interaction was no longer significant (*p* = 0.05).

^c^ Separate analysis due to significant interaction with FHR–SZ and FHR–BP risk groups (*p* < 0.001 for both). When analyzed separately, the interaction remained significant (*p* = 0.01).

### Does attachment at age 7 predict mental disorder at age 11?

Adjusted for presence of mental disorder at age 7, interaction was found for two of the attachment constructs; between the FHR‐SZ group and the security construct and between the FHR‐BP group and the disorganization construct. These were run in separate models (denoted *b* and *c* in Table 4). The security construct did not explain variance to significant levels when assessed as a main effect. The SSAP level of disorganization at age 7 significantly predicted higher prevalence of age 11 mental disorder although the variance explained by disorganized attachment at age 7 was little (Table 4). The SSAP insecurity construct did not predict mental disorder.

**TABLE 4:** General linear model presenting the degree to which child attachment at age 7 is predictive of child psychopathology at age 11.

	Type III sum of squares	df	Mean square	*F*	Significance	Partial eta squared
Risk group	1.58	2	0.79	4.87	**0.01**	0.02
Attachment insecurity construct at age 7^a^	0.30	1	0.30	1.86	0.17	<0.01
Presence of axis 1 diagnosis at age 7	15.02	1	15.02	92.29	**<0.001**	0.18
Total	125.00	419	–	–	–	–
Corrected total	87.71	418	–	–	–	–
Risk group	0.45	2	0.23	1.41	0.25	0.01
Risk group × attachment security construct at age 7^a^ ^,^ ^b^	1.02	2	0.51	3.16	**0.04**	0.02
Attachment security construct at age 7^a^	0.34	1	0.34	2.10	0.15	0.01
Presence of axis 1 diagnosis at age 7	15.97	1	15.97	99.59	**<0.001**	0.20
Total	125.00	419				
Corrected total	87.71	418				
Risk group	0.87	2	0.44	2.75	0.07	0.01
Risk group × attachment disorganization construct at age 7^a^ ^,^ ^c^	1.30	2	0.65	4.08	**0.02**	0.02
Attachment disorganization construct at age 7^a^	1.60	1	1.60	10.03	**0.002**	0.02
Presence of axis 1 diagnosis at age 7	14.33	1	14.33	90.17	**<0.001**	0.18
Total	125.00	419				
Corrected total	87.71	418				

*Note:* Bold values indicate the significant values at the 0.05 level.

^a^ Assessed with the Story Stem Attachment Profile (SSAP).

^b^ Separate analysis due to significant interaction with FHR–SZ group (*p* < 0.001).

^c^ Separate analysis due to significant interaction with FHR–BP group (*p* = 0.01).

## DISCUSSION

First, as hypothesized, we found no overall differences between control and high risk groups in neither attachment script content nor length. Nor did the dichotomous classification (secure vs. non‐secure attachments scripts) render any group differences. Second, we found evidence of continuity in attachment patterns from middle childhood to pre‐adolescence, as high levels of attachment security predicted later secure attachment. Third, we found parental functioning in middle childhood to be predictive of attachment in pre‐adolescence.

Our findings of non‐differential attachment are in line with previous findings of attachment representations in the same cohort when the children were 7 years of age (Gregersen et al., 2023), and with findings from other studies of 0–2.5‐aged children and ≥13 year‐old children of parents with psychosis or bipolar disorder (Doucette et al., 2013; Sameroff et al., 1982). Our findings contradict a smaller study of children of parents with bipolar disorder aged 12–17 which found elevated risks of dismissing attachment (Erkan et al., 2015), denoting feeling love‐worthiness but having negative expectations to other people and avoiding close relationships to protect oneself from disappointment (Bartholomew & Horowitz, 1991). Our findings also partly contradict a systematic review with limited data beyond 13 months of age which found some evidence of increased attachment disorder prevalence among schizophrenia offspring (Davidsen et al., 2015). Differences in age at assessment and contextual factors such as parental and socioeconomic circumstances may account for these disparate results. Notably, only the present study considered relations to friends at age 11, as they become increasingly important for attachment representations as the child reaches adolescence (Kerns et al., 1996, 2006).

It has previously been estimated that around two‐thirds of children in the general population have a secure attachment pattern (Van Ijzendoorn et al., 1999), however, data from children in middle childhood and pre‐adolescence in a Nordic context indicate secure attachment in about 56% of participants (Psouni et al., 2020). In addition, studies have shown that the number of children with a secure attachment pattern falls to approximately one‐third in populations experiencing adversity in terms of poverty and subsequently parental stress, challenges in the home environment, and child abuse (Weinfield et al., 2004). Although not entirely comparable, several such risk factors were found in our cohort and thus, differential attachment, with higher scores indicating richer secure base/safe haven in the control group and lower scores particularly in the FHR‐SZ group, was expected. The fact that scores were not lower among high risk groups may be partly due to selection bias in our cohort, although these were modest (Krantz et al., 2022), but they may however play a role particularly regarding attachment since families found in national registers, that were eligible for the study but did not participate, havebeen subject to more concern reports to the municipality and have children more frequently placed out of home (Krantz et al., 2022), compared with participants. Other possible reasons for the non‐differential findings may relate to successful provision of social support for both the family and the child (Table 1), that is, confounding by indication, as discussed elsewhere (Krantz et al., 2021), since support was provided for a substantial proportion of children particularly in the FHR‐SZ and FHR‐BP groups (Krantz et al., 2021).

Keeping limitations concerning comparisons of attachment constructs in mind, we found evidence of continuity in attachment, as security at age 7 predicted secure attachment script scores at age 11, denoting the stability of attachment over time, as previously found particularly concerning the security dimension (Opie et al., 2021). Significant interaction was seen with both risk groups. The lack of predictive findings from insecurity and disorganization to age 11 attachment security score may relate to sample size and represent a possible type 2 error since the means and ranges of these constructs were smaller than that of the security construct (Gregersen et al., 2023). They are however also in line with meta‐analytic evidence of higher longitudinal stability for secure, compared to insecure, attachment (Pinquart & Gerke, 2019). We also expected that the level of stimulation and support provided at home would explain variance in attachment security at age 11. The fact that such link was not found may again relate to confounding since external support is often provided to the most vulnerable families in ways which most likely will improve attachment (Krantz et al., 2021).

We found that parental level of functioning at age 7 predicted age 11 child attachment score. This finding is in line with a previous study which found that the severity and chronicity of the parental disorder as well as the parental socio‐economic status had a higher impact on the child's attachment than the diagnosis of the parent (Sameroff et al., 1982). Adjusting for age 7 mental disorder, we found that a higher age 7 disorganization construct score predicted higher prevalence of mental disorder at age 11, in line with our cross‐sectional findings at age 7 (Gregersen et al., 2023) and previous findings of a link between non‐secure attachment and risk of mental disorders (Brumariu & Kerns, 2010; Fearon et al., 2010; Madigan et al., 2013; Sroufe, 2005).

The findings of possible relationships between parental functioning and attachment, and between disorganized attachment and mental disorder, are not surprising. Secure attachment scripts imply parent‐child interactions where the child's distress, difficulties and negative affect were likely met with sensitivity, the child received support, was soothed, and helped to return to a more positive affect state and exploration (Psouni & Apetroaia, 2014). Parental functioning can affect capacity to such sensitivity and support. Previous studies have shown that this support boosts the child's capacity to regulate emotions, and through this regulation, moderates the risk for mental disorder, and have further shown that this is particularly true among children of parents with severe mental illness (Doucette et al., 2013).

### Strengths and limitations

A major strength of the VIA 11 Study is that it is a large, nationwide, population‐based prospective cohort of same‐aged pre‐adolescent children of parents with schizophrenia or bipolar disorder and a matched population‐based control group, all assessed in multiple domains of relevance for mental development. The study had well validated tests of mental disorder, functioning, and level of stimulation and support in the home, making it possible to examine predictors related to attachment across two time points in a time period where studies on attachment is sparse and of particular relevance in children at risk of mental illness as the prevalence of such disorder increases markedly as the children approach puberty (Mendle et al., 2020). Further, the study had well educated and trained assessors and scoring team members, and the attrition rate in our study was low.

Although necessary to secure age‐appropriate assessment, using different attachment assessment tools at age 7 and 11 is a substantial limitation, particularly given the lack of studies on validity of the SSAP and its combined use with the SBST. The SSAP is more suitable for children up to age 9, while the SBST is suitable for children from 8 years of age and onwards. This complicates comparisons since the inter‐assessment reliability between these instruments has, to our knowledge, not been tested previously. This is, to our knowledge, the first study to combine the SSAP (at age 7) and SBST (at age 11), and results indicate high concordance between the measures, providing some further evidence of construct validity for the SBST and some first evidence of construct validity for the SSAP. While we chose in our study to mainly focus on predictors in a manner where one attachment measure and another measure, for example, functioning or mental disorder, were included, we also found it relevant to briefly examine SSAP and SBST dimensions, and these results should be interpreted with caution, while replication and further validation is warranted.

Finally, fewer children than expected belonging to the PBC (control) group told stories that indicated a secure base script. This is perhaps reflecting a conservative scoring regime, or assessment conditions which were not perceived as sufficiently comfortable for the children to produce their stories while they were being recorded. Much effort was made to make the participating children feel safe and comfortable during assessment, but some children could have been affected by the research setting.

## CONCLUSION

Our study has some important clinical implications: It indicates that awareness of the need for support in case of low levels of functioning may be appropriate in mental health settings in order to prevent low parental functioning from influencing negatively on their child's attachment pattern. Further, in child and adolescent psychiatric mental health settings it is relevant to be aware that difficulties with early age attachment, in particular with disorganized attachment, may indicate being at risk of mental disorder in adolescence. Supporting attachment in mental health settings when relevant may decrease prevalence of child psychopathology, and our findings support the relevance for new intervention studies in this field (Bikic et al., 2022).

To our knowledge, this is the first longitudinal study of attachment representations, based on a large and nationwide prospective cohort of same‐aged early adolescents of parents diagnosed with schizophrenia or bipolar disorder, and matched controls. Our findings add to the literature concerning attachment patterns in offspring of parents with severe mental disorder, its relation to child mental disorder and particularly adds knowledge to the time period between middle childhood and early adolescence which has only been covered in few studies (Davidsen et al., 2015; Doucette et al., 2013; Erkan et al., 2015; Zahn‐Waxler et al., 1984).

## AUTHOR CONTRIBUTIONS

**Mette Falkenberg Krantz**: Conceptualization; data curation; formal analysis; investigation; methodology; project administration; writing – original draft; writing – review & editing. **Maja Gregersen**: Conceptualization; data curation; methodology; supervision; validation; writing – review & editing. **Lotte Veddum**: Data curation; investigation; writing – review & editing. **Carsten Hjorthøj**: Formal analysis; methodology; supervision; validation; writing – review & editing. **Åsa Kremer Prøsch**: Data curation; investigation; writing – review & editing. **Jessica Ohland**: Data curation; project administration; writing – review & editing. **Julie Marie Brandt**: Data curation; writing – review & editing. **Sinnika Birkehøj Rohd**: Data curation; investigation; writing – review & editing. **Christina Bruun Knudsen**: Data curation; investigation; writing – review & editing. **Anna Krogh Andreasen**: Data curation; investigation; writing – review & editing. **Nicoline Hemager**: Data curation; formal analysis; investigation; methodology; project administration; supervision; validation; writing – review & editing. **Aja Greve**: Data curation; investigation; methodology; project administration; supervision; writing – review & editing. **Ole Mors**: Investigation; project administration; writing – review & editing. **Merete Nordentoft**: Conceptualization; data curation; formal analysis; funding acquisition; investigation; methodology; project administration; validation; writing – review & editing. **Elia Psouni**: Conceptualization; formal analysis; investigation; methodology; supervision; validation; writing – review & editing. **Anne A. E. Thorup**: Conceptualization; formal analysis; funding acquisition; investigation; methodology; project administration; supervision; validation; writing – review & editing.

## CONFLICT OF INTEREST STATEMENT

The authors declare no conflicts of interest.

## ETHICAL CONSIDERATIONS

The study was approved by the local ethical committee and the Data Protection Agency (Thorup et al., 2018). Written consent was obtained from legal guardians upon oral and written information.

## Supporting information

Supporting Information S1

## Data Availability

Data sharing is limited in accordance with Danish GDPR legislation but can be considered upon reasonable request from other researchers and approval from relevant authorities.
