# Supplementary material for: Attachment representations in pre‐adolescents at familial high risk of schizophrenia or bipolar disorder and population‐based controls—Characteristics of attachment from middle childhood to pre‐adolescence, and its relation to parental functioning and child mental disorder
Source: JCPP Adv. 2024 Sep 12;5(3):e12274. doi: 10.1002/jcv2.12274 (PMC12446683; doi:10.1002/jcv2.12274)
Supplement: Supplementary file 1 — Supporting Information S1 [file JCV2-5-e12274-s001.docx]

Supplementary tables and figures

Supplementary Table 1a: Internal consistency (1a) and interrater reliability (1b) for the Secure Base Script Test.

1a)

The story “Accident”:

| Cronbach’s alpha | Cronbach’s alpha based on standardized items | N of items |
| --- | --- | --- |
| 0.96 | 0.96 | 2 |

The story ”Moving”

| Cronbach’s alpha | Cronbach’s alpha based on standardized items | N of items |
| --- | --- | --- |
| 0.96 | 0.96 | 3 |

The story “Trouble at School”

| Cronbach’s alpha | Cronbach’s alpha based on standardized items | N of items |
| --- | --- | --- |
| 0.96 | 0.96 | 2 |

The story ”Math Test”

| Cronbach’s alpha | Cronbach’s alpha based on standardized items | N of items |
| --- | --- | --- |
| 0.97 | 0.97 | 3 |

1b)

|  | Intraclass correlation | 95% CI, lower bound | 95% CI, upper bound | Value | F Test with true Value 0, df 1 | F Test with true Value 0, df 2 | Sig |
| --- | --- | --- | --- | --- | --- | --- | --- |
| Single measures | 0.89 | 0.83 | 0.93 | 24.25 | 49 | 98 | <0.001 |
| Average measures | 0.96 | 0.93 | 0.98 | 24.25 | 49 | 98 | <0.001 |

Supplementary Figure 1 – Flowchart:

Data extracts from Danish National Registries^a^ of children with FHR-SZ^b, c^, FHR-BP^d^ and 10 PBCs^e^ for each case, born between 01-09-04 to 31-08-09

**(24, 706)**

Children who were too old at start of study 01-01-2013, or too young by VIA 7 inclusion end date 31-01-16

**(5, 376)**

Children retrieved as matched controls to FHR-BP

**(7, 373)**

N=5 376

Children eligible for inclusion in VIA 7^f^

**(11, 957)**

PBC

**(10, 110)**^g^

FHR-BP

**(774)**

FHR-SZ

**(1, 073)**

No contact attempted **(663)**^h^

No contact attempted **(9 ,791)**

No contact attempted (**560)**

Attempted contact

**(319)**

Attempted contact

**(214)**

Attempted contact

**(410)**

Non-respondents **(40)** Declined **(54)**

Non-respondents **(40)** Declined **(79)**

Non-respondents **(92)** Declined **(116)**

FHR-SZ included in VIA 7

**(202)**

PBC included in VIA 7

**(200)**

FHR-BP included in VIA 7

**(120)**

Declined (**23**)

Declined **(19)**

Declined **(15)**

PBC children included in VIA 11:

**(181)**

PBC children who participated in SSAP assessment **(186)**

PBC children who participated in SBST assessment **(160)**

FHR-BP children included in VIA 11:

**(105)**

FHR-BP children who participated in SSAP assessment **(113)**

FHR-BP children who participated in SBST assessment **(92)**

FHR-SZ children included in VIA 11:

**(179)**

FHR-SZ children who participated in SSAP assessment **(183)**

FHR-SZ children who participated in SBST assessment **(157)**

**^a^ Danish National Registries:** Danish Civil Registration System and Danish Psychiatric Central Research Register. Based on the Danish Psychiatric Central Research Register, adults with a diagnosis of schizophrenia spectrum psychotic illness, (defined as schizophrenia, delusional disorder or schizoaffective disorder, ICD 10-codes: F20, F22 and F25 or ICD 8-codes: 295, 297, 298.29, 298.39, 298.99), or with a diagnosis of bipolar disorder (ICD 10-code F30, F31 or ICD 8-codes: 296.19 296.39) were identified. In-patient contacts could be any time between April 1, 1969, when the register was established, and the end of 2011. Out-patient contacts are registered from January 1, 1995 and onwards.

**^b^ FHR-SZ:** Children of parents with schizophrenia spectrum disorders.

**^c^ Double diagnosed parents:** Parents with diagnoses of schizophrenia and bipolar disorder were assigned to the schizophrenia high risk group in accordance with the ICD-10 hierarchy.

**^d^ FHR-BP:** Children of parents with bipolar disorder.

**^e^ PBC:** Population-based control children of parents with no diagnoses of schizophrenia spectrum disorders or bipolar disorder.

**^f^ Research protection:** As of May 2011, legislation was enacted to protect individuals´ phone numbers from being called for participation in scientific research. Therefore, there were eligible children who were not contacted and enrolled in VIA 7.

**^g^ Controls selection:** Up to 10 controls were retrieved for each child in the schizophrenia spectrum disorder group and the bipolar disorder group. Controls were matched to cases on gender, municipality and exact age. The original intent was to only select control cases that were matched to children in the schizophrenia familial high-risk group. However, there are 38 BP-controls among the 200 total controls.

**^h^ Definition of contact:** First through letters sent to the child´s address. If the family did not respond, contact by telephone was attempted (calls and text messages), if a phone number could be found.

^i^ **Flowchart has previously been reported in** (Krantz, 2021)

Supplementary Text:

*Internal consistency and interrater reliability*

High internal consistency over the four outlines has previously been reported (.87 (Psouni & Apetroaia, 2014), and .77 (Psouni et al., 2015)) as well as high construct and concurrent validity, measured against self-reported attachment security (Kerns et al., 2001) and an attachment interview (Steele & Steele, 2005), see (Psouni & Apetroaia, 2014). High interrater reliability for the SBST has been previously reported (.85- .95 in (Psouni & Apetroaia, 2014), .85 in (Psouni et al., 2015)).

*Dropout analyses*

A total of 409 children (FHR-SZ=157, FHR-BP=92 and PBC=160, mean (SD) age 11.9 (0.23)) participated in the age 11 SBST assessment (Table 1 and Supplementary Figure 1). Participants from the total VIA 11 cohort who did not participate in the SBST assessment neither differed from those who participated in the SBST assessment in terms of age or sex, nor did those who participated in the SBST assessment differ regarding age 7 attachment constructs concerning disorganization or security but did however differ concerning the insecurity construct (Supplementary Table 2). Thus, the mean (SD) for the SSAP insecurity construct for those who participated in SBST was 1.02 (0.78) versus 1.14 (0.79) for those who did not participate in SBST.

*Inspection of data and outlier characterization*

Through stem-and-leaf plots which were used to assess extreme values of age 11 attachment, we identified 15 children across risk groups with the highest (richest secure base content) (5.0-6.0, mean 5.4) and lowest (most sparse secure base content) (1.6-2.5, mean 2.2) values, respectively. All outliers were considered as valid upon inspection and kept in the dataset. All risk groups were equally represented for both highest and lowest values, with no significant differences across groups. The children with very high scores had lower prevalence of psychiatric diagnoses (N (%)=7 (46.7) at age 7 and N (%) = 8 (53.3) at age 11), compared with children with low scores (N (%) = 8 (53.3) at age 7 and N (%)= 11 (73.3) at age 11). Also, their parents had higher levels of functioning (mean (SD)= 83.13 (23.82) at age 7 and 78.47 (14.3) at age 11)), compared with children with low scores (mean (SD)=76.60 (14.35) at age 7 and 75.67 (16.49) at age 11), and a lower proportion had received municipality support for the child (N (%)= 6 (40.0)) or family (N (%)= 2 (13.3)), compared with children with low scores (N (%)= 8 (53.3) for the child *as well as* for the family). Further, no children had been placed away from their biological parents, compared to <5 among children with low scores, and fewer lived with a single caregiver (N (%)= 4 (26.7), compared with children with low scores (N (%)= 5 (33.3)). Also, the age 7 level of stimulation and support provided in the child’s home was higher (mean (SD)=48.20 (4.75) compared with 46.87 (5.28) for children with low attachment scores), and the children’s level of functioning was higher among children with high attachment scores (mean (SD)=78.60 (10.95)), compared with children with low scores (mean (SD)=57.6 (15.26)) (data not shown).

*Word count estimates in SBST and correlation between age 7 and 11 assessments of attachment*

A strong and positive correlation was found at the SBST age 11 assessment between the number of words used by the child for the four base script stories and the total score (p<0.001) (Supplementary Table 3). Likewise, a strong and positive correlation was found between the SSAP security construct at age 7 and the SBST total score at age 11 (p<0.001) (Supplementary Table 4).

*Associations between placement out of home and attachment*

In order to examine selection bias of relevance for attachment, we were able to exploratively examine associations between placement within participants and attachment. At age 7, we thus found more disorganization (mean (SD) 1.15 (1.49) among the children as compared to those living with their biological parents (0.53 (0.97)), p=0.009, Pearson’s R 0.175 (data not shown), indicating that a larger group of FHR children would likely have higher disorganization construct scores if no selection bias had been present in our cohort.

Supplementary Table 2: Age 11 Secure Base Script Test dropout stratified by child’s sex, age, and age 7 attachment score.

| Participated in SBST VIA 11: |  |
| --- | --- |
| Yes, N (%) | 409 (88) |
| No, N (%) | 56 (12) |
| Dropout by sex of the child^a^: |  |
| Pearson Chi-Square: | 0.25 |
| Value (df) | 1.32 (1) |
| Dropout by age of the child^a^: |  |
| Pearson Chi-Square: | 0.26 |
| Value (df) | 250.95 (237) |
| Dropout by attachment construct at age 7^b^ – disorganization construct |  |
| Pearson Chi-Square: | 0.443 |
| Value (df) | 33.51 (33) |
| Dropout by attachment construct at age 7^b^ – insecurity construct |  |
| Pearson Chi-Square: | **0.03** |
| Value (df) | 48.74 (32) |
| Dropout by attachment construct at age 7^b^ – security construct |  |
| Pearson Chi-Square: | 0.54 |
| Value (df) | 51.40 (53) |
| Dropout by global functioning at age 7^b^: |  |
| Pearson Chi-Square: | 0.39 |
| Value (df) | 60.30 (58) |
| Dropout by problem behavior at age 7^b^: |  |
| Pearson Chi-Square: | 0.208 |
| Value (df) | 86.84 (77) |

^a^ As compared with overall cohort participation rate at age 11.

^b^ As compared with SBST age 11 participation.

Supplementary Table 3: Correlation between number of words used in the Secure Base Script Test (SBST) and SBST score at age 11.

|  |  | Total secure base script score | Total word count for all base script stories |
| --- | --- | --- | --- |
| Total secure base script score | Pearson correlation | 1 | 0.349* |
|  | Sig. (2-tailed) |  | **<0.001** |
|  | N | 409 | 405 |

*Correlation is significant at the 0.01 level (2-tailed).

Supplementary Table 4: Correlation analysis between the Story Stem Attachment Profile (SSAP) base script constructs at age 7 versus the Secure Base Script Test (SBST) score at age 11.

| Total secure base script score at age 11 | | | |
| --- | --- | --- | --- |
|  | Pearson correlation | Sig (2-tailed) | N |
| SSAP security construct at age 7 | 0.29 | **<0.001** | 381 |
| SSAP insecurity construct at age 7 | 0.02 | 0.75 | 381 |
| SSAP disorganization construct at age 7 | -0.04 | 0.47 | 381 |

|  |
| --- |

Kerns, K. A., Aspelmeier, J. E., Gentzler, A. L., & Grabill, C. M. (2001). Parent-child attachment and monitoring in middle childhood. *Journal of Family Psychology*. https://doi.org/10.1037/0893-3200.15.1.69

Krantz, M. F. et al. (2021). Home environment of 11-year-old children born to parents with schizophrenia or bipolar disorder – a controlled, 4-year follow-up study: The Danish High Risk and Resilience Study – VIA 11. *Psychological Medicine*, 1–11. https://doi.org/DOI: 10.1017/S0033291721004487

Psouni, E., & Apetroaia, A. (2014). Measuring scripted attachment-related knowledge in middle childhood: The Secure Base Script Test. *Attachment and Human Development*. https://doi.org/10.1080/14616734.2013.804329

Psouni, E., Di Folco, S., & Zavattini, G. C. (2015). Scripted secure base knowledge and its relation to perceived social acceptance and competence in early middle childhood. *Scandinavian Journal of Psychology*. https://doi.org/10.1111/sjop.12208

Steele, H., & Steele, M. (2005). The Construct of Coherence as an Indicator of Attachment Security in Middle Childhood: The Friends and Family Interview. In *Attachment in middle childhood.*
